# Infrared Image Super-Resolution Network Utilizing the Enhanced Transformer and U-Net

**DOI:** 10.3390/s24144686

**Published:** 2024-07-19

**Authors:** Feng Huang, Yunxiang Li, Xiaojing Ye, Jing Wu

**Affiliations:** School of Mechanical Engineering and Automation, Fuzhou University, Fuzhou 350108, China; huangf@fzu.edu.cn (F.H.); 17864285690@163.com (Y.L.); yexiaojingyxj@163.com (X.Y.)

**Keywords:** transformer, generative adversarial network, infrared image, image super-resolution

## Abstract

Infrared images hold significant value in applications such as remote sensing and fire safety. However, infrared detectors often face the problem of high hardware costs, which limits their widespread use. Advancements in deep learning have spurred innovative approaches to image super-resolution (SR), but comparatively few efforts have been dedicated to the exploration of infrared images. To address this, we design the Residual Swin Transformer and Average Pooling Block (RSTAB) and propose the SwinAIR, which can effectively extract and fuse the diverse frequency features in infrared images and achieve superior SR reconstruction performance. By further integrating SwinAIR with U-Net, we propose the SwinAIR-GAN for real infrared image SR reconstruction. SwinAIR-GAN extends the degradation space to better simulate the degradation process of real infrared images. Additionally, it incorporates spectral normalization, dropout, and artifact discrimination loss to reduce the potential image artifacts. Qualitative and quantitative evaluations on various datasets confirm the effectiveness of our proposed method in reconstructing realistic textures and details of infrared images.

## 1. Introduction

Infrared imaging utilizes infrared detectors to capture images, differentiating target objects from the background based on the disparities found in radiated heat. This technique effectively addresses the constraints encountered in visible imaging, such as limited object penetration and varying weather conditions, thus playing a pivotal role in diverse fields including climate research [1], disaster warning [2], rescue [3] and resource exploration [4]. However, the resolution of infrared images depends on the quantity and size of the dedicated infrared detector array. The hardware limitation can restrict the imaging quality.

Image super-resolution (SR) can transform low-resolution (LR) images into high-resolution (HR) ones without enhancing optical detector sensitivity or developing complex optical imaging systems, significantly reducing costs. Recent advancements in deep learning have demonstrated exceptional performance in utilizing SR for image enhancement.

Early deep learning-based image SR methods (e.g., SRCNN [5], VDSR [6], and EDSR [7]) rely on convolutional neural network (CNN) to learn the mapping from LR images to HR images. However, since convolutional kernels can only perceive local information from the input image, it is necessary to stack deep networks to gradually enlarge the receptive field. Therefore, CNN may be limited in handling global information in images. Recently, transformer-based methods [8,9,10,11,12] have become widely popular in the field of image SR. They leverage a self-attention mechanism to effectively capture long-range dependencies and handle multi-scale features in images, thus achieving better SR performance. Despite this, the above methods rely only on custom degradation models, such as bicubic interpolation (BI), for paired training, which tend to overlook the complex degradation processes present in real-world images, resulting in overly smooth SR results.

Nowadays, blind-image SR networks (e.g., BSRGAN [13] and Real-ESRGAN [14]) have gained attention for their ability to handle real-world SR tasks. These methods generate training pairs using unknown degradations, which leads to more robust and generalizable models that better meet real-world requirements. However, these works primarily focus on visible images. Few studies have extended image SR to the field of infrared images.

To address the SR challenge in infrared images, we design the Residual Swin Transformer and Average Pooling Block (RSTAB) and construct the novel Infrared Image Super-Resolution model based on Swin Transformer [8] and Average Pooling [15], SwinAIR. SwinAIR combines the strengths of CNN and transformer, allowing for the sufficient extraction of features from infrared images. To achieve SR reconstruction of real infrared images, we further integrate the SwinAIR with the generative adversarial network (GAN) and build the SwinAIR-GAN, which can recover realistic textures and features matching the real infrared images. The primary contributions of this study are as follows:

- We explore a novel infrared image SR reconstruction model, SwinAIR. We introduce the Residual Swin Transformer and Average Pooling Block into the deep feature extraction module of SwinAIR. This configuration effectively extracts both low- and high-frequency infrared features while concurrently fusing them. Comparative results with existing methods demonstrate that our model exhibits superior performance in the SR reconstruction of infrared images.
- We combine SwinAIR with the U-Net [16] to construct SwinAIR-GAN, further exploring the problem of SR reconstruction for real infrared images. SwinAIR-GAN utilizes SwinAIR as the generator network and employs U-Net as the discriminator network. Comparative results with similar methods demonstrate that our model can generate infrared images with better visual effects and more realistic features and details.
- We incorporate spectral normalization, dropout, and artifact discrimination loss to minimize possible artifacts during the restoration process of real infrared images and enhance the generalization ability of SwinAIR-GAN. We also expand the degradation space of the degradation model to emulate the degradation process of real infrared images more accurately. These improvements enable the generation of infrared images that closely align with the textures and details of real-world images while avoiding over-smoothing.
- We establish an infrared data acquisition system that can simultaneously capture LR and HR infrared images of corresponding scenes, addressing the issue of lacking reference images in the real infrared image SR reconstruction task.

The rest of this paper is structured as follows. Section 2 provides a brief overview of related studies, and Section 3 offers a detailed description of the proposed network. In Section 4, we validate the effectiveness of our network by testing it on various datasets and conducting ablation experiments. Finally, the conclusions are presented in Section 5.

## 2. Related Works

Infrared image SR methods are derived from visible image SR methods. In this section, we briefly review the image SR reconstruction research status related to our work.

### 2.1. Traditional Infrared Image Super-Resolution Reconstruction Methods

Traditional infrared image SR methods, similar to visible image SR methods, can be categorized into frequency domain-based, dictionary-based, and other methods. Frequency domain-based methods decompose the infrared image into the spatial domain and the frequency domain [17], processing the image separately in each domain. For example, Choi et al. [18] distinguished the edge pixels of infrared images and utilized visible images as guidance to enhance the high-frequency information of infrared images. Mao et al. [19] transformed the infrared image SR problem into a sparse signal reconstruction problem based on differential operations, significantly enhancing the algorithm’s speed. Dictionary-based methods [20,21,22,23,24,25] focus on constructing the mapping relationship between LR and HR image patches. By leveraging the relationships between image patches, these methods retain the pattern information of LR images in HR images. This type of method has garnered researchers’ attention for its interpretability. Other traditional methods include iterative methods [26,27], regularization methods [28], and so on.

With the advancement of deep learning technology, transformer and GAN technologies have begun to be widely applied in the field of image SR reconstruction, demonstrating significant advantages.

### 2.2. Transformer-Based Image Super-Resolution Reconstruction Methods

Transformer was initially proposed by Vaswani et al. [29] to address sequence modeling in natural language processing tasks. The self-attention mechanism allows the transformer to effectively capture global dependencies. Additionally, its parallel computing capability accelerates training and enhances efficiency. With the development of vision [30,31], detection [32,33], and video-vision [34,35] transformers, superior results have been achieved by transformers in various visual tasks.

In the field of image SR reconstruction, there are mainly two types of transformer-based frameworks. The first type utilizes the transformer structure entirely, such as the end-to-end network proposed by Chen et al. [9]. However, this framework may lack adaptability to biases in some cases. The second type combines transformer with CNN, leveraging the strengths of CNN in capturing local features and transformer in modeling global dependency relationships. This integration enables better capture of both local details and global structures in the image. For example, Liu et al. [8] introduced a backbone Swin Transformer that replaces the standard multi-head self-attention with a shifted-window multi-head self-attention for dense hierarchical feature map prediction, enabling efficient interactions among non-overlapping local windows while addressing the challenges posed by larger-scale images and huge training datasets. Liang et al. [10] built upon this idea and proposed SwinIR, which has demonstrated impressive performance in various tasks, including image SR and dehazing. Subsequently, researchers have continuously produced new research results [11,12] with better performance based on this foundation. Cao et al. [36] took SwinIR as the architecture and proposed the CFMB-T model for infrared remote sensing images SR reconstruction. Yi et al. [37] further utilized a hybrid convolution–transformer network for feature extraction, and the suggested HCTIR deblur model has made great progress in infrared image deblurring.

Despite the promising performance of transformer-based methods in image SR reconstruction, strategies that combine the transformer with CNN for deep feature extraction are still limited. This limitation often results in networks overlooking high-frequency information present in images. The problem is particularly pronounced when dealing with infrared images that involve blurring and a lack of high-frequency details. To address this issue, we propose SwinAIR, which better integrates the transformer with CNN to effectively capture both low- and high-frequency information, thereby enhancing the SR reconstruction capability for infrared images.

### 2.3. GAN-Based Image Super-Resolution Reconstruction Methods

GAN was proposed by Goodfellow et al. in 2014 [38]. It consists of a generator network and a discriminator network that are trained adversarially. The generator network progressively creates more realistic samples, while the discriminator network continuously refines its ability to differentiate between real and artificial images. Training concludes when the discriminator network struggles at a given threshold to distinguish between the two, resulting in high-quality generated samples.

Ledig et al. [39] first applied the GAN to image SR reconstruction and introduced the SRGAN. The SRGAN incorporates perceptual loss, which provides more detailed and lifelike SR images. Subsequently, Yan et al. [40] optimized the image quality assessment approach and used it to improve the loss function of SRGAN, further enhancing its performance. Wang et al. [41] introduced the ESRGAN model in 2018, which incorporates a residual dense block into its generator network and employs a relative discriminator network. By enhancing perceptual loss and introducing interpolation, ESRGAN achieves exceptional perceptual reconstruction effects. Based on the ESRGAN, several newer networks have been developed, including BSRGAN [13], RFB-ESRGAN [42], and Real-ESRGAN [14].

Liu et al. [43] introduced the natural image gradient prior. They used visible images from corresponding scenes as guidance to generate infrared images with improved subjective visual quality. Huang et al. [44] proposed HetSRWGAN, which incorporates heterogeneous convolution and adversarial training. The reconstructed infrared images using this method achieve higher objective evaluation metrics. They also introduced the lightweight PSGAN [45] using a multi-stage transfer learning strategy. Liu et al. [46] designed a generator network with recursive attention modules, significantly improving the SR quality of vehicle infrared images. Lee et al. [47] proposed the Style Transfer Super-Resolution Generative Adversarial Network, STSRGAN, enhancing the quality of LR infrared images containing very small targets.

Although the aforementioned GAN-based methods have shown high potentiality, their training processes are often unstable and result in unintended artifacts. To generate more realistic infrared images, we employ the spectral normalization-based U-Net [16] as the discriminator network and add the artifact discrimination loss function in our SwinAIR-GAN.

## 3. Proposed Method

In this section, we will comprehensively introduce the SwinAIR and SwinAIR-GAN along with their technical details.

### 3.1. SwinAIR

#### 3.1.1. Overall Structure

**Figure 1:**
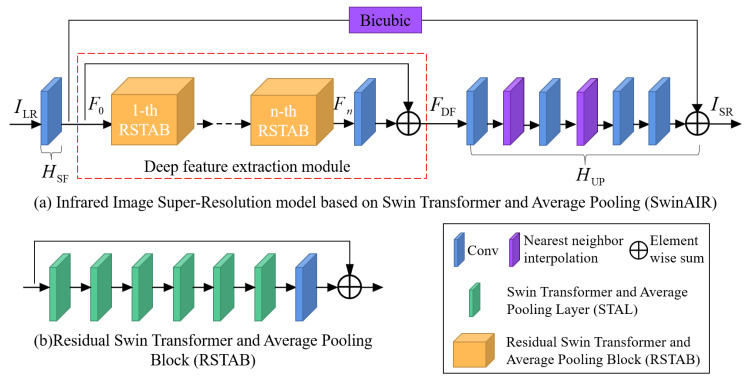
Structure of our proposed Infrared Image Super-Resolution model based on Swin Transformer and Average Pooling, SwinAIR. Firstly, the input LR infrared $I_{\mathrm{LR}}$ undergoes the shallow feature extraction module $H_{\mathrm{SF}}$ to extract the shallow feature map $F_{0}$; then, $F_{0}$ is deep extracted and refined by the deep feature extraction module. We obtain $F_{n}$ after n RSTABs. After $F_{n}$ undergoes convolutional operations, it is further combined with $F_{0}$ through residual connection to obtain the deep feature map $F_{\mathrm{DF}}$. Finally, $F_{\mathrm{DF}}$ is input into the upsampling module $H_{\mathrm{UP}}$ to generate the output SR infrared image $I_{\mathrm{SR}}$. The interpolation operation here is bicubic.

The structure of SwinAIR is displayed in Figure 1. Infrared images generally have lower resolution and contain more low-frequency information than visible images. Therefore, the input LR infrared image first undergoes shallow feature extraction and is passed directly to the end of the deep feature extraction network. This helps to better preserve its low-frequency information, thereby improving the performance of SwinAIR.

Concurrently, the shallow features are input to the deep feature extraction module to obtain the deep features. We adopt the hierarchical design of the deep feature extraction module from the Swin Transformer [8]. This structure allows for the extraction and integration of features at different scales in the infrared image, helping the network to better learn critical information and reduce noise.

Ultimately, the shallow features and deep features are merged and fed into the upsampling module to generate the SR infrared image.

In the shallow feature extraction module, the LR infrared image is processed by a 3 × 3 convolutional layer to extract shallow feature information as follows:

(1) $$F_{\mathrm{SF}}\;=\;H_{\mathrm{SF}}\left(I_{\mathrm{LR}}\right)$$

where $I_{\mathrm{LR}}$ denotes the LR infrared image, and $I_{\mathrm{LR}}\in R^{H_{L}\times W_{L}\times C_{L}}$, $H_{L}$, $W_{L}$, and $C_{L}$ indicate the height, width, and channel number of the LR infrared image, respectively. $H_{\mathrm{SF}}\left(•\right)$ represents the shallow feature extraction module, and $F_{\mathrm{SF}}$ represents the shallow feature map.

In the deep feature extraction module, multiple stacked RSTABs (as shown in Figure 1b) and a 3 × 3 convolutional layer are employed to further extract deep features. Residual connections are also used for feature aggregation to obtain deep features, and this process can be simplified as

(2) $$F_{\mathrm{DF}}\;=\;H_{\mathrm{DF}}\left(F_{\mathrm{SF}}\right)$$

where $H_{\mathrm{DF}}\left(•\right)$ corresponds to the deep feature extraction module, and $F_{\mathrm{DF}}$ represents the deep feature map. The deep feature extraction module not only expands the receptive field by stacking convolutional layers but also combines the advantages of both transformer and CNN to effectively extract low- and high-frequency image information. With local and global residual learning, the problem of gradient degradation in deep networks is mitigated, significantly reducing the parameters and overall training time.

Finally, the feature map obtained from the deep feature extraction module is reconstructed layer by layer according to a specified upsampling factor that follows the order of the upsampling module (as shown in Figure 1a). Simultaneously, the upsampled LR infrared image is moved to the end of the upsampling module, where it is added to the reconstructed feature map to produce the final SR infrared image:

(3) $$I_{\mathrm{SR}}\;=\;H_{\mathrm{UP}}\left(F_{\mathrm{DF}}\right)$$

where $H_{\mathrm{UP}}\left(•\right)$ represents the upsampling module, $I_{\mathrm{SR}}$ is the SR infrared image, and $I_{\mathrm{SR}}\in R^{H_{S}\times W_{S}\times C_{S}}$, $H_{S}$, $W_{S}$, and $C_{S}$ indicate the height, width, and channel number of the SR infrared image, respectively (similar to $I_{\mathrm{LR}}$). The specific steps of the upsampling reconstruction process can be expressed as

(4) $$F_{\mathrm{UP}}\;=\;H_{C}\left(D\left(H_{C}\left(H_{\mathrm{Near}}\left(H_{C}\left(H_{\mathrm{Near}}\left(H_{C}\left(F_{\mathrm{DF}}\right)\right)\right)\right)\right)\right)\right)$$

(5) $$I_{\mathrm{SR}}\;=\;F_{\mathrm{UP}}+H_{\mathrm{Bicubic}}\left(F_{\mathrm{SF}}\right)$$

where $H_{C}\left(•\right)$ denotes a 3 × 3 convolutional layer, $H_{\mathrm{Near}}\left(•\right)$ represents the nearest neighbor interpolation, $H_{\mathrm{Bicubic}}\left(•\right)$ denotes the bicubic interpolation, $F_{\mathrm{UP}}$ represents the reconstructed feature map and D(•) signifies the dropout operation. Dropout is a regularization technique originally proposed to address overfitting issues in classification networks. A recent study has demonstrated that dropout can enhance the generalizability of SR networks [48]. Therefore, we apply dropout before the last convolutional layer in the upsampling module in SwinAIR with a probability of 0.5. The effectiveness of this approach is confirmed by ablation experiments in Section 4.4.2.

#### 3.1.2. Deep Feature Extraction Module

Drawing inspiration from previous work [8,49], we develop the deep feature extraction module for SwinAIR. It is mainly composed of several RSTABs, which combine the strengths of transformer and CNN to effectively extract low- and high-frequency information from infrared images.

Specifically, the deep feature extraction module consists of *n* RSTABs and a 3 × 3 convolutional layer with residual connections. The intermediate feature map obtained from the ith RSTAB, $F_{i}$, and the final output deep feature map, $F_{\mathrm{DF}}$, are given as follows:

(6) $$F_{i}\;=\;H_{\mathrm{RSTA}B_{i}}\left(F_{i-1}\right),i=1,2,\ldots ,n$$

(7) $$F_{\mathrm{DF}}\;=\;H_{C}\left(F_{n}\right)+F_{0}$$

where $F_{0}$ denotes the shallow feature map $F_{\mathrm{SF}}$, and $H_{\mathrm{RSTA}B_{i}}\left(•\right)$ represents the ith RSTAB. By introducing a convolutional layer at the end of the deep feature extraction module, the inductive bias of convolutional operations is incorporated into the transformer, providing a more robust foundation for the subsequent aggregation of shallow and deep features [10].

As illustrated in Figure 1b, each RSTAB is composed of six Swin Transformers and Average Pooling Layers (STALs) and a 3 × 3 convolutional layer with the residual connection. This allows local features to be transmitted and fused across different layers, enabling the network to deeply learn the mapping relationship between LR and HR infrared images, thereby accelerating the network’s learning and convergence speed.

For the given input feature map of the ith RSTAB, $F_{i,0}$, the intermediate output feature map can be further defined as follows:

(8) $$F_{i,j}\;=\;H_{\mathrm{STA}L_{i,j}}\left(F_{i,j-1}\right),j=1,2,\ldots ,m$$

(9) $$F_{i}\;=\;H_{C_{i}}\left(F_{i,m}\right)+F_{i,0}$$

where $H_{\mathrm{STA}L_{i,j}}\left(•\right)$ denotes the jth STAL in the ith RSTAB, and $F_{i,j}$ represents the feature map output in the jth STAL of the ith RSTAB. $H_{C_{i}}\left(•\right)$ represents the final 3 × 3 convolutional layer in the ith RSTAB.

**Figure 2:**
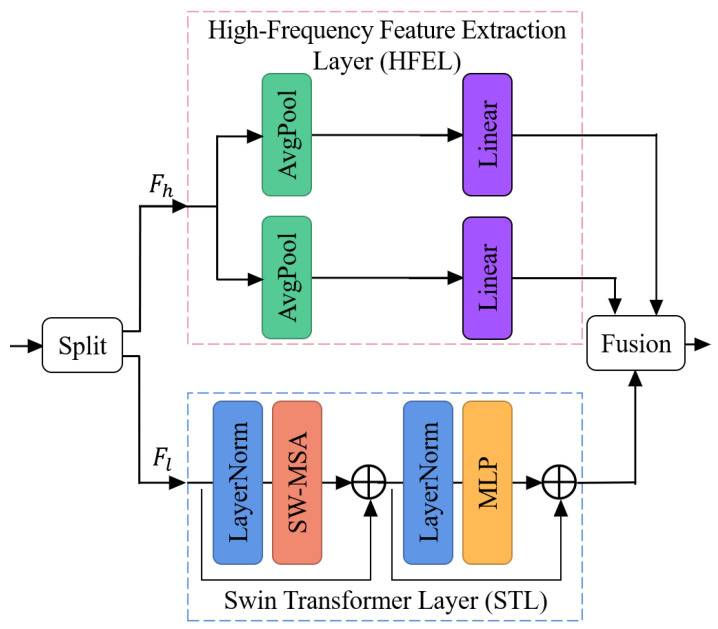
Structure of the Swin Transformer and Average Pooling Layer (STAL). There are two ways to combine CNN with the transformer: serial or parallel. The serial method means that each layer can process either low- or high-frequency information but not both. Therefore, to allow each layer to process both types of information simultaneously, a parallel structure with channel separation is used to integrate the CNN and transformer. The feature map is initially divided into $F_{h}$ and $F_{l}$, which are then separately fed into the High-Frequency Feature Extraction Layer (HFEL) and the Swin Transformer Layer (STL). In STL, SW-MSA and MLP represent the self-attention module based on the shift-window mechanism and the multi-layer perceptron module, respectively.

The internal structure of STAL is shown in Figure 2. Each STAL has two sections: the High-Frequency Feature Extraction Layer (HFEL), which consists of two parallel average pooling and fully connected layers, and the Swin Transformer Layer (STL) [8]. The use of channel separation strategies allows the network to independently process low- and high-frequency information in infrared images, mitigating interference during the learning of different structural features. This approach also reduces computational complexity and accelerates the convergence of the network.

Specifically, for the jth STAL of the ith RSTAB, we assume the input feature map $F_{i,j-1}\in R^{H_{i,j-1}\times W_{i,j-1}\times C_{i,j-1}}$. $H_{i,j-1}$, $W_{i,j-1}$, and $C_{i,j-1}$ indicate the height, width, and channel number of $F_{i,j-1}$. $F_{i,j-1}$ is partitioned channel-wise to $F_{h}\in R^{H_{h}\times W_{h}\times C_{h}}$ and $F_{l}\in R^{H_{l}\times W_{l}\times C_{l}}$ ($C_{h}=C_{i,j-1}/3,C_{l}=2C_{i,j-1}/3$); then, $F_{h}$ and $F_{l}$ are independently fed to the HFEL and the STL.

With the sensitivity of the average pooling layers and the ability of the fully connected layers to capture details [49], the HFEL comprises two parallel paths to perform high-frequency information extraction, each containing an average pooling layer and a fully connected layer. $F_{h}$ is divided into two channel-wise maps $F_{h1}\in R^{H_{h}\times W_{h}\times \left(C_{h}/2\right)}$ and $F_{h2}\in R^{H_{h}\times W_{h}\times \left(C_{h}/2\right)}$. $F_{h1}$ and $F_{h2}$ are separately processed in the parallel paths to generate their respective outputs ${\hat{F}}_{h1}$, ${\hat{F}}_{h2}$ as follows:

(10) $${\hat{F}}_{h1}=FC\left(Avgpool\left(F_{h1}\right)\right)$$

(11) $${\hat{F}}_{h2}=FC\left(Avgpool\left(F_{h2}\right)\right)$$

where Avgpool(•) signifies the average pooling layer and FC(•) symbolizes the fully connected layer.

To decrease the computational burden associated with the standard transformer for image processing, we use STL for low-frequency feature extraction. As shown in Figure 2, STL primarily consists of the self-attention module based on the shift-window mechanism (SW-MSA) and the multi-layer perceptron module (MLP). SW-MSA calculates multi-head self-attention within local regions of the image by shift window. This process captures the correlations between different local regions of the image, thereby extracting local structures and texture information. MLP combines non-linear transformations to better capture the semantic information of the image. For $F_{l}$, the processing process can be described as shown below:

(12) $${\hat{F}}_{l}=H_{\mathrm{MLP}}\left(LN\left(H_{\mathrm{MSA}}\left(LN\left(F_{l}\right)\right)+F_{l}\right)\right)+H_{\mathrm{MSA}}\left(LN\left(F_{l}\right)\right)+F_{l}$$

where ${\hat{F}}_{l}$ represents the feature map after processing by the STL. LN(•) denotes the layer normalization operation, $H_{\mathrm{MSA}}$ represents the SW-MSA module, and $H_{\mathrm{MLP}}$ represents the MLP module.

Finally, the low-frequency information feature map after processing by the STL, ${\hat{F}}_{l}$, and the high-frequency information feature maps after processing by the HFEL, ${\hat{F}}_{h1}$, ${\hat{F}}_{h2}$, are fused along the channel dimension to get $F_{i,j}$, which can be represented as

(13) $$F_{i,j}=FU\left({\hat{F}}_{h1},{\hat{F}}_{h2},{\hat{F}}_{l}\right)$$

where FU(•) represents the fusion operation.

#### 3.1.3. Loss Function

SwinAIR uses L1 loss as the loss function.The L1 loss calculates the pixel-wise differences between SR and HR images as follows:

(14) $$L_{1}=\frac{1}{hwc}\sum_{i,j,k}\left|I_{i,j,k}^{\mathrm{SR}}-I_{i,j,k}^{\mathrm{HR}}\right|$$

where *h*, *w*, and *c* denote the height, width, and channel number of the image, respectively. $I_{i,j,k}^{\mathrm{SR}}$ and $I_{i,j,k}^{\mathrm{HR}}$ represent the pixel values of the SR and HR images at the spatial location, $\left(i,j,k\right)$, respectively.

### 3.2. SwinAIR-GAN

**Figure 3:**
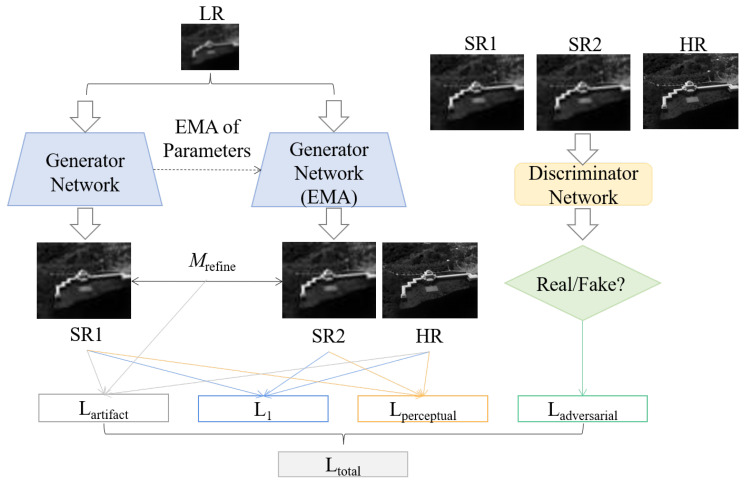
SwinAIR-GAN method. The generator network generates the SR infrared feature map SR1 from the LR infrared image and applies an exponential moving average (EMA) to the parameters to obtain the infrared feature map SR2. Various loss functions are then computed for SR1, SR2, and the HR infrared image. Finally, the corresponding discriminator network judge the authenticity of the generated infrared image.

To generate infrared images that more closely align with real physical characteristics, we further design SwinAIR-GAN based on SwinAIR. The proposed SwinAIR-GAN structure for real infrared image SR is depicted in Figure 3. We use SwinAIR as the generator network and a spectral normalization-based U-Net as the discriminator network.

#### 3.2.1. Generator Network

The generator network utilizes our proposed SwinAIR, which effectively extracts and reconstructs information from infrared images. Detailed information about SwinAIR can be found in Section 3.1.

#### 3.2.2. Discriminator Network

**Figure 4:**
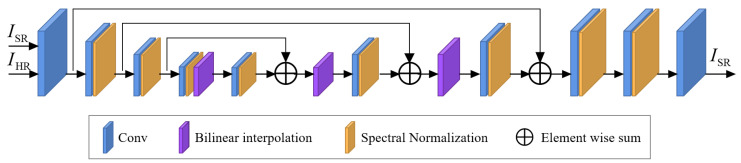
Structure of the discriminator network. We adopt a U-Net structure based on spectral normalization.

The discriminator network (as shown in Figure 4) employs a U-Net architecture. However, the complex degradation space and structure can increase training instability. To mitigate this risk, spectral normalization [50] is used to stabilize training. This approach can also alleviate the introduction of unnecessary artifacts during GAN training, thereby achieving a balance between local image detail enhancement and artifact suppression.

The discriminator network primarily comprises 10 convolutional layers, 8 spectral normalization layers, and 3 bilinear interpolation layers. Each of the first 9 convolutional layers is followed by a Leaky ReLU activation function. Table 1 provides the detailed information about each convolutional layer in the discriminator network.

#### 3.2.3. Loss Function

Appropriate loss functions are crucial to quantifying the differences between SR and HR images and optimizing network training. Conventional networks use a combination of per-pixel, perceptual, and adversarial losses in real-image SR tasks. To enhance image details and eliminate artifacts, the SwinAIR-GAN incorporates additional artifact discrimination loss. Below, we will discuss these different loss functions in more detail.

L1 Loss. The information about L1 loss can be found in Section 3.1.3.

Perceptual Loss. In contrast to L1 loss, perceptual loss aims to make SR infrared images more visually similar to HR infrared images rather than just minimizing pixel differences. This process is expressed as follows:

(15) $$L_{\mathrm{perceptual}}=\frac{1}{h_{l}w_{l}c_{l}}\sqrt{{\sum_{i,j,k}\left(\phi_{i,j,k}^{l}\left(I_{\mathrm{SR}}\right)-\phi_{i,j,k}^{l}\left(I_{\mathrm{HR}}\right)\right)}^{2}}$$

where $h_{l}$, $w_{l}$, and $c_{l}$ denote the height, width, and channel numbers of the lth feature map, respectively. $\phi_{i,j,k}^{l}\left(I_{\mathrm{SR}}\right)$ and $\phi_{i,j,k}^{l}\left(I_{\mathrm{HR}}\right)$ represent the pixel values of the SR and HR feature maps extracted from the lth layer of the pretrained network at the spatial location $\left(i,j,k\right)$, respectively.

Adversarial Loss. Adversarial loss is primarily used to generate realistic SR images. The adversarial losses of SwinAIR-GAN are represented as follows:

(16) $$L_{\mathrm{adversarial}\_G}=-\log D_{\theta }\left(I_{\mathrm{SR}}\right)$$

(17) $$L_{\mathrm{adversarial}\_D}=-\log D_{\theta }\left(I_{\mathrm{HR}}\right)-\log\left(1-D_{\theta }\left(I_{\mathrm{SR}}\right)\right)$$

where $L_{\mathrm{adversarial}\_G}$ and $L_{\mathrm{adversarial}\_D}$ represent the adversarial losses of the generator network and discriminator network, respectively. $D_{\theta }\left(I_{\mathrm{SR}}\right)$ and $D_{\theta }\left(I_{\mathrm{HR}}\right)$ denote the probabilities the SR and HR images are trained to be classified as real images, respectively.

Artifact Discrimination Loss. Artifact discrimination loss is used to minimize SR image artifacts [51] as follows:

(18) $$L_{\mathrm{artifact}}={∥M_{\mathrm{refine}}\cdot \left(I_{\mathrm{HR}}-I_{\mathrm{SR}1}\right)∥}_{1}$$

where $I_{\mathrm{SR}1}$ refers to the SR image obtained through the generator network, and $M_{\mathrm{refine}}$ represents the refined feature map.

Overall loss function. Taking the aforementioned losses into account, the comprehensive loss function of the SwinAIR-GAN is as follows:

(19) $$L_{\mathrm{total}}=\alpha \cdot L_{1}+\beta \cdot L_{\mathrm{perceptual}}+\gamma \cdot L_{\mathrm{adversarial}}+\delta \cdot L_{\mathrm{artifact}}$$

where α, β and γ represent the proportionate contributions of each loss type (i.e., $L_{1}$, perceptual, adversarial and artifact discrimination types) to the comprehensive loss function. Based on experience, we set α=1, β=1, γ=0.1 and δ=1.

#### 3.2.4. Degradation Model

**Figure 5:**
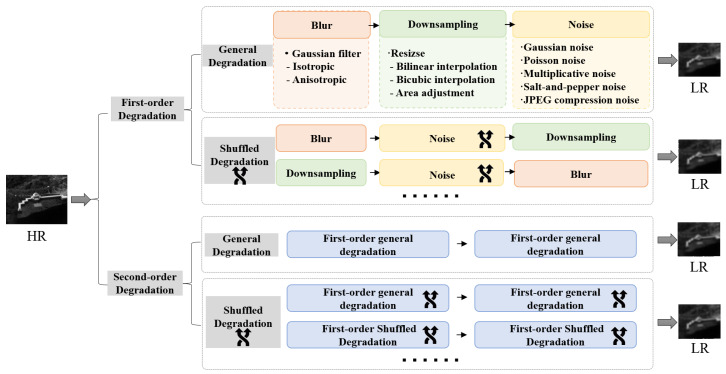
The degradation model. We expand the degradation space and consider first- and second-order degradation processes to emulate the real degradation of infrared images more accurately.

Classical SR networks commonly employed bicubic downsampling to simulate image degradation. However, this approach has difficulty dealing with complex and unknown degradations in real infrared images. Issues such as blurring and ghosting are commonplace. To tackle this problem, we extend the degradation space (as shown in Figure 5) to more effectively simulate the process real infrared image degradation.

Typically, image degradation can be represented by a general model, such as the following:

(20) $$I_{\mathrm{LR}}=\left(I_{\mathrm{HR}}⊗k\right){↓}_{s}+n_{\delta },\left\{k,s,\delta \right\}\in R$$

where *k* denotes the blur kernel, ⊗ denotes the convolution operation, ↓ denotes the down-sampling operation, *s* is the scaling factor, and $n_{\delta }$ represents noise (typically additive white Gaussian) with a standard deviation of δ.

To expand the degradation space, we consider both first- and second-order degradations. For the first-order degenerate process, general and shuffled degradations are applied. The general degradation refers to the sequential application of blur, downsampling, and noise in sets of two or three. The shuffled degradation involves the random ordering of the degradation steps. For the second-order degenerate process, the same two degradation types are considered. The general degradation comprises two first-order degradation processes, while the shuffled degradation involves randomly shuffling blur, downsampling, and noise for each of the two first-order degradation processes.

In the degradation model, blur, noise, and downsampling are the three key factors affecting image degradation. By expanding the degradation space of these factors, true degradation processes are approximated to improve the applicability and generalizability of the network. Our proposed degradation model considers these key factors that affect the degradation of infrared images as follows:

Blur. In our model, isotropic and anisotropic Gaussian blur kernels are used. A Gaussian blur kernel $k\left(i,j\right)$ with a kernel size of 2t+1 can be represented as follows:

(21) $$k\left(i,j\right)=\frac{1}{N}\exp\left(-\frac{1}{2}C^{T}\sum^{-1}C\right),C={\left[i,j\right]}^{T}$$

where ∑ denotes the covariance matrix, *C* represents the spatial coordinates, and *N* is a normalization constant, $\left(i,j\right)\in \left[-t,t\right]$, and is sampled from a Gaussian distribution. ∑ can be further expressed as

(22) $$\sum =\left[\begin{array}{cc} \cos\theta & -\sin\theta \\ \sin\theta & \cos\theta \end{array}\right]\left[\begin{array}{cc} \mu_{1}^{2} & 0\\ 0 & \mu_{2}^{2} \end{array}\right]\left[\begin{array}{cc} \cos\theta & \sin\theta \\ -\sin\theta & \cos\theta \end{array}\right]$$

where $\mu_{1}$ and $\mu_{2}$ represent the standard deviation along two main axes (i.e., the eigenvalues of the covariance matrix), and θ is the rotation angle. When $\mu_{1}=\mu_{2}$, $k\left(i,j\right)$ is an isotropic Gaussian blur kernel. Otherwise, $k\left(i,j\right)$ is an anisotropic Gaussian blur kernel.

The Gaussian blur kernel configurations are hyperparameters. Different settings affect the blurring of infrared images during degradation and the complexity of the model. Based on previous works (e.g., BSRGAN [13] and Real-ESRGAN [14]) and the characteristics of infrared images, we experiment with various settings combinations and ultimately determine the optimal settings.

For the kernel size, we uniformly sample from [7×7,9×9,…,21×21] to obtain varying degrees of blurring. For the isotropic Gaussian kernel, we uniformly sample the standard deviation from [0.1,2.8]. For the anisotropic Gaussian kernel, we uniformly sample the standard deviation from [0.5,6] and [0.5,8] with equal probability, and we uniformly sample the rotation angle from [0,π] to simulate motion blurring at different angles.

By randomly using these Gaussian kernels with different configurations during the degradation, we achieve a variety of complex blurring effects that closely simulate the degradation process of real infrared images, thus effectively optimizing the performance of SwinAIR-GAN.

Noise. The noise in infrared images can usually be classified into two categories: random noise and fixed pattern noise. Random noise includes two types of stationary random white noise: Gaussian noise and Poisson noise. Fixed pattern noise is primarily characterized by image non-uniformity and blind elements. Non-uniform and scanning-array blind elements contribute to multiplicative noise, whereas staring-array blind elements result in salt-and-pepper noise. Therefore, we consider four types of noise for processing: Gaussian noise, Poisson noise, multiplicative noise, and salt-and-pepper noise. We also include JPEG compression noise to expand the degradation space.

Gaussian noise is random noise with a probability density function (PDF) that follows a Gaussian distribution. The PDF of Gaussian noise is given by

(23) $$p\left(x\right)=\frac{1}{\sqrt{2\pi }\sigma }e^{-{\left(x-\mu \right)}^{2}/2\sigma^{2}}$$

where *x* represents the grayscale value of the image noise, μ represents the mean value of *x*, and σ represents the standard deviation of *x*.

Poisson noise follows a Poisson distribution, and its intensity is proportional to that of the image. The noises at different pixel points are independent of one another. The PDF of Poisson noise is given by

(24) $$p\left(x=m\right)=\frac{\lambda^{m}e^{-\lambda }}{m!},m=0,1,2,\cdots$$

where λ is the expectant value, and λ>0.

Multiplicative noise is produced by random variations in channel characteristics and is related to the signal through multiplication. This type of noise follows a rayleigh or gamma distribution. In infrared images, the multiplicative noise caused by the non-uniformity is numerous and mixed together in a unified form. In a focal plane detector array, the response of each detection element to a mixture of various non-uniform factors can be expressed as follows:

(25) $$x_{i}=a_{i}\times I+b_{i},i=1,2,\cdots ,M$$

where *x* represents the response of the detection element, *I* represents the incident infrared radiation, and *M* represents the number of detection elements in the array. The non-uniformity of each detection element is influenced by the combined impact of the gain $a_{i}$, and offset, $b_{i}$, of each unit.

Salt-and-pepper noise is caused by changes in signal pulse intensity. It includes two variations: high-intensity salt-like noise represented by white dots and low-intensity pepper-like noise represented by black dots. Typically, they both occur simultaneously. Dieickx et al. [52] suggested that the blind elements of a staring array manifest as salt-and-pepper noise. The PDF of salt-and-pepper noise in infrared images can be represented as follows:

(26) $$P\left(x\right)=\left\{\begin{array}{c} P_{a},x=a\\ P_{b},x=b\\ 0,\mathrm{other} \end{array}\right\}$$

where $P_{a}$ and $P_{b}$ represent the density distributions of pepper noise and salt noise, respectively.

JPEG compression is a widely used lossy compression technique for digital images, which reduces storage and transmission requirements by discarding unimportant components. The quality of the compressed image is determined by the quality factor *q* ($q\in \left[0,100\right]$). A higher q value corresponds to a lower compression ratio and better image quality. When *q* > 90, the image quality is considered great, and there will be no obvious artifacts introduced. In our degradation model, the range for the JPEG quality factor is set to within [30,95].

Downsampling. Downsampling is a fundamental operation used to change the image size and generate LR images. There are four interpolation methods for image resizing: nearest neighbor, bilinear, bicubic, and area adjustment. Nearest neighbor interpolation introduces pixel deviation issues [53]; hence, we focus primarily on the other three.

The different resizing methods have their own effects on images. Our degradation model randomly selects from downsampling, upsampling, and maintaining the original size to diversify the degradation space. Assuming that the scaling factor is *s*, the image size is first changed by *a* ($a\in \left[1/2,s\right]$), resulting in an intermediate-sized image. After degradation, the image is resized by a scaling factor of s/a, causing the image size to match the downsampling scope required by the original image.

## 4. Experimental Details and Results

In this section, we provide a detailed introduction to the experimental details and validation results of SwinAIR and SwinAIR-GAN. Both quantitative and qualitative analyses demonstrate the superiority of our proposed method for infrared image SR reconstruction.

### 4.1. Datasets and Metrics

Training sets. Due to the scarcity of high-quality public infrared image datasets available for training, we utilize visible image datasets as the training set based on previous work [45]. Specifically, we use the DV2K [54] (DIV2K [55] + Flickr2K [7]) dataset, which contains 3450 images rich in texture and detail.

Test sets. To maximize the generalization of test scenarios and thoroughly validate the effectiveness of the proposed method, we employ six datasets as the test sets for SwinAIR: the CVC-14 dataset [56], the Flir dataset [57], the Iray universal infrared dataset [58] (Iray-384), the Iray infrared maritime ship dataset [59] (Iray-ship), the Iray infrared aerial photography dataset [60] (Iray-aerial photography), and the Iray infrared security dataset [61] (Iray-security). The CVC-14 dataset [56], the Flir dataset [57], and the Iray-384 dataset [58] mainly consist of road traffic scene images, while the Iray-ship dataset [59], the Iray-aerial photography dataset [60], and the Iray-security dataset [61] consist of maritime ship scenes, aerial street scenes, and security surveillance scenes, respectively. For SwinAIR-GAN, we use the ASL-TID dataset [57], the Iray-384 dataset [58] and a self-built dataset as the test sets. The ASL-TID dataset [57] primarily includes scenes with pedestrians, cats and horses, and our self-built dataset comprises 138 images covering various scenes such as buildings, roads, vehicles and pedestrians.

**Figure 6:**
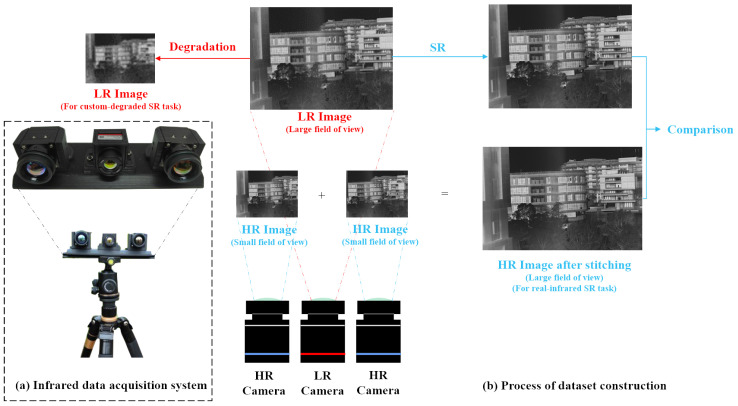
Our infrared data acquisition system. (**a**) System hardware. (**b**) Process of dataset construction. For SR tasks with custom degradation, images captured by the Iray camera are used as HR reference images and to calculate metrics. Due to differences in images captured by different cameras, we use no-reference metrics for the quantitative analysis of SR reconstruction quality for real SR tasks. The HR images captured by the Jinglin Chengdu cameras are stitched together to serve as only reference images for real SR tasks, assessing whether the SR reconstructed images are consistent with real textures and details.

The purpose of creating our own dataset is to address the challenge of lacking reference images in real infrared image SR tasks, which makes it difficult to thoroughly compare the texture and detail recovery effects of different methods. Specifically, we build an infrared data acquisition system consisting of an LR Iray infrared camera and two HR Chengdu Jinglin infrared cameras. The parameters and models of the cameras are detailed in Table 2. The specific process of acquiring infrared image data using the above-mentioned system is shown in Figure 6. We place three cameras parallel to each other with the LR Iray infrared camera in the middle and the two HR Chengdu Jinglin infrared cameras on the sides. Different degradation methods are applied to infrared images captured by the Iray camera across various scenes to construct LR–HR image pairs (in the custom-degraded SR task, the infrared images captured by the Iray camera are considered as HR images relative to the degraded images). Simultaneously, we perform parallax correction on the images captured by the two Chengdu Jinglin infrared cameras and then stitch them together to obtain images with a higher resolution than the original ones captured by the Iray camera. These higher-resolution images will be used as reference images in real infrared SR tasks (i.e., direct SR reconstruction of images captured by the Iray camera) to evaluate the effectiveness of different methods in reconstructing details and textures from unknown degraded infrared images.

Evaluation metrics. To quantitatively evaluate the SR performance of our proposed method, we use full-reference image quality evaluation metrics, peak signal-to-noise ratio (PSNR), and structural similarity (SSIM) for SwinAIR. To further evaluate the realism of the generated infrared images of SwinAIR-GAN, we also use non-reference image quality evaluation metrics, natural image quality evaluator (NIQE), and perceptual index (PI), in addition to PSNR and SSIM. All evaluation metrics are computed on the luminance channel after converting the reconstructed images to the YCbCr color space. We assess the efficiency of SwinAIR using floating-point operations (FLOPs) and inference speed.

### 4.2. Model and Training Settings

Model settings. For SwinAIR, the number of RSTABs (*n*) and the number of STALs (*m*) are set to 6. The window size of the SW-MSA in STL is 8 with the number of attention heads set to 6. The weights for the high-frequency channel ($F_{h}$) and the low-frequency channel ($F_{l}$) are set to 1/3 and 2/3, respectively. For SwinAIR-GAN, the degradation model parameters are detailed in Section 3.2.4. The generator network parameters are the same as those of SwinAIR, and the discriminator network settings are provided in Table 1.

Training settings. We use the Adam method to optimize our SwinAIR and SwinAIR-GAN, with the three hyperparameters of Adam, $\beta_{1}$, $\beta_{2}$, and ε, set to 0.9, 0.999, and $10^{-8}$, respectively. The initial learning rate is set to $2\times 10^{-4}$ and decays using a multi-step strategy. The number of iterations for model updates is set to $6\times 10^{5}$. All trainings are implemented in PyTorch and conducted on a server equipped with 8 Nvidia A100 GPUs with a batch size of 32. The input LR image size is set to 64 × 64. During the training process, the dataset is augmented using seven data augmentation techniques, including random horizontal flipping, random vertical flipping, and random 90° rotations.

### 4.3. SwinAIR

#### 4.3.1. Comparisons with the State-of-the-Art Methods

We compare our SwinAIR method with existing mainstream classical image SR methods on multiple datasets to evaluate their performance in reconstructing infrared images. All LR images are degraded using bicubic downsampling.

Quantitative Evaluations. We evaluate the SR reconstruction performance of SwinAIR and other methods on infrared images across six datasets at different scales. The quantitative comparison results are shown in Table 3. Our network achieves superior reconstruction performance with fewer parameters. At the ×2 and ×3 scales, our method achieves the best metrics across all datasets except for the CVC-14 dataset [56], where the results are second best. At the ×4 scale, our method achieves the best performance across all test sets while reducing the parameters by 19% compared to SwinIR. We also compare the efficiency of SwinAIR with several well-performing methods at the ×4 scale, and the results are shown in Table 4. The FLOPs and inference speed measurements are performed on an NVIDIA A100 GPU with the input image size set to 64×64. To ensure the accuracy of the inference speed measurement, we first perform 50 warm-up inferences, which are followed by 100 inferences to calculate the average inference time. Compared to CNN-based methods (e.g., EDSR [7] and SRFBN [62]), transformer-based methods (e.g., SwinIR [10], HAT [12] and SwinAIR) achieve better performance at the cost of reduced inference speed. Nonetheless, the use of the shift-window mechanism can effectively reduce their computational complexity. Additionally, due to the implementation of channel separation strategies, SwinAIR achieves the lowest computational complexity among the compared methods.

Visual comparison. We compare the visual reconstruction results of different methods at the ×4 scale on different datasets. Compared with other methods, our proposed SwinAIR demonstrates superior visual effects and is able to better restore accurate details and textures.

**Figure 7:**
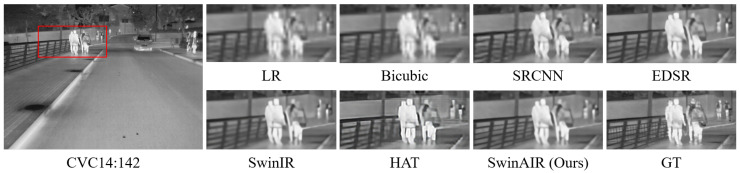
Visual comparison results achieved by different methods on the CVC14 dataset [56] at the ×4 scale. GT represents the original HR image in the red box of the leftmost image. Our proposed method can restore more realistic fence details.

**Figure 8:**
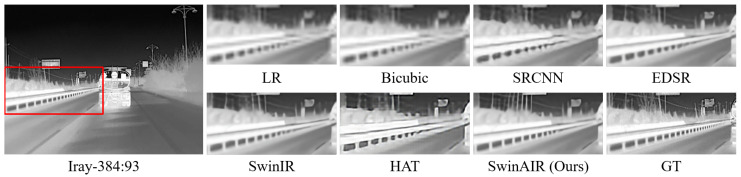
Visual comparison results achieved by different methods on the Iray-384 dataset [58] at the ×4 scale. GT represents the original HR image in the red box of the leftmost image. Our proposed method can restore more realistic fence details.

**Figure 9:**
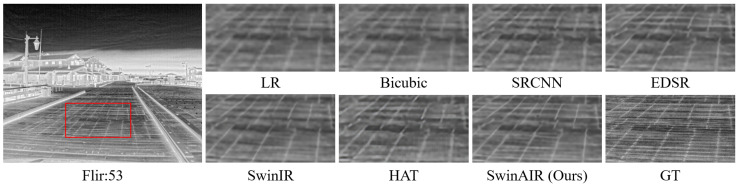
Visual comparison results achieved by different methods on the Flir dataset [57] at the ×4 scale. GT represents the original HR image in the red box of the leftmost image. Our proposed method can restore closely spaced ground textures.

**Figure 10:**
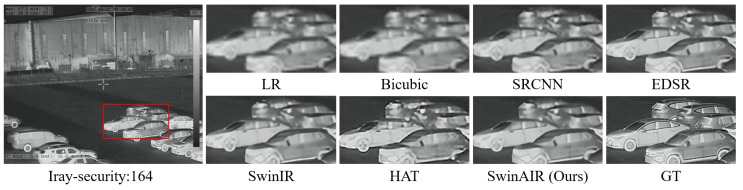
Visual comparison results achieved by different methods on the Iray-security dataset [61] at the ×4 scale. GT represents the original HR image in the red box of the leftmost image. Our proposed method can restore more realistic vehicle contours.

**Figure 11:**
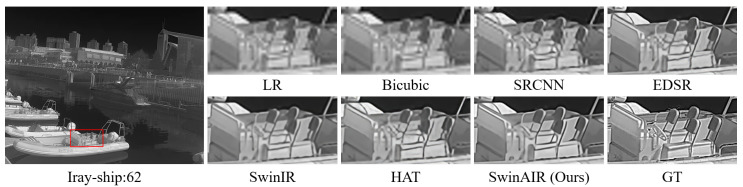
Visual comparison results achieved by different methods on the Iray-ship dataset [59] at the ×4 scale. GT represents the original HR image in the red box of the leftmost image. Our proposed method can restore more realistic ship contours.

**Figure 12:**
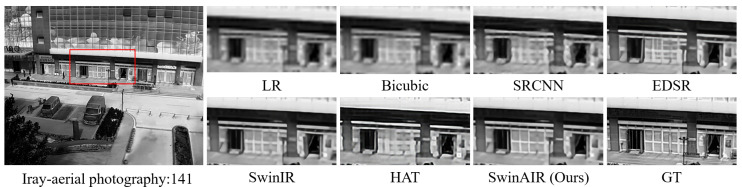
Visual comparison results achieved by different methods on the Iray-aerial photography dataset [60] at the ×4 scale. GT represents the original HR image in the red box of the leftmost image. Our proposed method can restore more realistic lines of door frames.

For instance, in the restoration of fence details (Figure 7 and Figure 8), bicubic, SRCNN [5], and EDSR [7] produce visually less appealing blurry images. HAT [12] generates overly sharpened images with speckled and serrated textures. SwinIR [10] and SwinAIR perform better overall, but SwinIR [10] introduces distortions and twists at intersections of distant fence lines. Only SwinAIR can reconstruct such detailed features consistently with reference images. In the restoration of ground textures (Figure 9), all methods except SwinAIR fail to accurately restore the closely spaced horizontal lines at the bottom. Similar situations can also be observed in the contours of cars and ships (Figure 10 and Figure 11), and the lines of door frames (Figure 12), where SwinAIR shows superior capability in recovering fine and contour features. This demonstrates that handling different frequency features separately can effectively enhance the network’s performance.

#### 4.3.2. Ablation Study

To further validate the effectiveness of each component in SwinAIR and the rationality of parameter selection, we conduct extensive ablation experiments. The models are retrained on the DV2K [54] dataset and reevaluated on the CVC14 dataset [56] at the ×4 scale.

**Figure 13:**
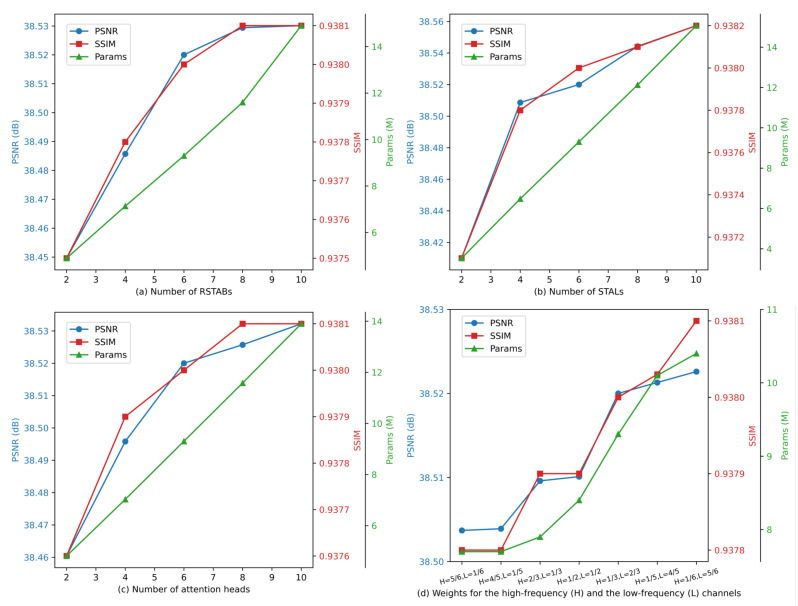
We evaluate the impact of different module configurations and parameter selections on the performance of SwinAIR using PSNR (dB), SSIM, and the number of parameters (M) as performance metrics. In the figures, params represent the number of parameters, H and L denote the weights of high-frequency and low-frequency channels, respectively. (**a**) The impact of the number of RSTABs on the performance of SwinAIR. (**b**) The impact of the number of STALs within each RSTAB on the performance of SwinAIR. (**c**) The impact of the number of attention heads in the STL on the performance of SwinAIR. (**d**) The impact of different weights of the high-frequency and the low-frequency channels on the performance of SwinAIR.

Number of RSTABs. Initially, as the number of RSTABs increases, the network’s ability to capture features continuously improves, and its performance steadily enhances. However, when the number of RSTABs increases further, the network performance gains begin to saturate while parameters continue to increase rapidly. The relation between network performance and parameters with varying RSTABs is shown in Figure 13a. To balance the performance and parameters of SwinAIR, we ultimately set the number of RSTABs to 6.

Number of STALs in RSTAB. As shown in Figure 13b, the performance of SwinAIR starts to plateau when the number of STALs reaches 4 and saturates when the number reaches 6. There is little difference in performance between having 6 and 8 STALs. Therefore, we ultimately set the number of STALs to 6.

Number of attention heads. Using more attention heads can enhance the network’s ability to capture complex features, but it also introduces additional computational overhead. By analyzing the impact of different numbers of attention heads on network performance, as shown in Figure 13c, we find that setting the number of attention heads to 6 achieves a good balance between performance and parameters.

Different weights of the high-frequency and the low-frequency channels. The selection of channel separation weights is related to the distribution of high-frequency and low-frequency information in infrared images. As shown in Figure 13d, we notice that as the weight of the low-frequency channel increases, the network performance continuously improves. This indicates that there is still a substantial amount of learnable low-frequency information in the images. However, due to the high complexity of the low-frequency feature processing structure (STL), the model’s parameters also increase accordingly. When the low-frequency channel weight exceeds 2/3, further increasing the weight provides minimal performance gain. Therefore, we ultimately set the high-frequency channel weight to 1/3 and the low-frequency channel weight to 2/3.

**Figure 14:**
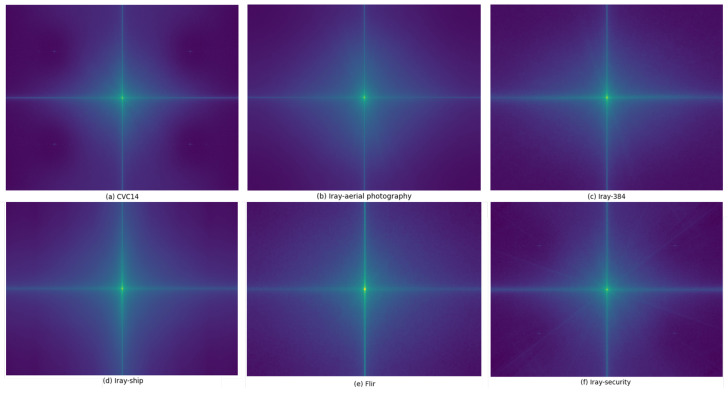
Frequency domain analysis of different datasets. We perform the Fourier transform and spectrum centralization on the images in the datasets. The central part of the spectrogram represents low-frequency information, while the edges represent high-frequency information. The overall range of the spectrum distribution is similar across different datasets.

Different datasets have varied devices, shooting environments, and objects, which may affect the optimal selection of channel separation weights. To further verify the reasonableness of channel separation weight settings, we perform frequency domain analysis on different datasets. Specifically, we conduct the Fourier transform on the images in each dataset and compute the average spectrum to represent the overall distribution of frequency information in the dataset. The comparison results are shown in Figure 14. Although the spectrograms of different datasets vary in detail, the overall range and intensity of frequency information distribution are similar. This demonstrates that the channel separation weights can be applied to different types of infrared images.

Different structures of the HFEL. Pooling layers in CNNs can reduce data dimensionality and extract high-frequency information from images. We validate the impact of using different pooling layers and different numbers of branches within the HFEL on SwinAIR’s performance. The results are shown in Table 5. The results indicate that the average pooling layer outperforms the max pooling layer. Additionally, the structure with two parallel pooling and fully connected layers significantly outperforms the single-branch structure. Interestingly, we find that using both average and max pooling layers together does not perform as well as using either one alone. We hypothesize that this is due to the mismatch and difficulty in fusing features extracted by different pooling layers. Therefore, we ultimately adopt a structure with two parallel average pooling and fully connected layers in the HFEL.

### 4.4. SwinAIR-GAN

#### 4.4.1. Comparisons with the State-of-the-Art Methods

We compare the SwinAIR-GAN method with other GAN-based image SR methods across multiple datasets to thoroughly evaluate its performance in restoring real infrared images. For the sake of fairness, we compare methods only with similar types of loss functions due to the strong correlation between evaluation metrics and the loss functions of GAN-based image SR methods [64].

Quantitative Evaluations. We evaluate the SR reconstruction performance of SwinAIR-GAN and other GAN-based methods on infrared images across three datasets. We validate the image restoration performance under both bicubic downsampling (BI degradation) and Gaussian blur downsampling (BD degradation) scenarios at the ×4 scale. The quantitative comparison results are shown in Table 6. Additionally, we perform the ×4 scale SR reconstruction directly on the images in the dataset to validate the SR reconstruction performance of different methods on unknown degraded real scenes. We use no-reference evaluation metrics, NIQE and PI, for quantitative analysis. The quantitative comparison results are shown in Table 7. Apart from achieving second-best results on the ASL-TID dataset [57] for unknown degraded real scenes, SwinAIR-GAN outperforms other models of the same category across all other datasets under various degradation conditions.

**Figure 15:**
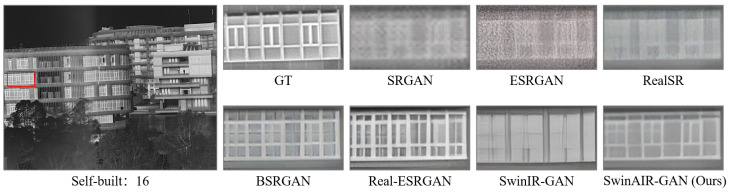
Visual comparison results achieved by different methods for real infrared images on the self-built dataset at the ×4 scale. GT represents the reference image in the red box of the leftmost image, which is obtained by stitching images from two HR cameras. We perform SR reconstruction on the corresponding real-scene images captured by the LR camera and observe the texture differences with the GT images to evaluate performance.

**Figure 16:**
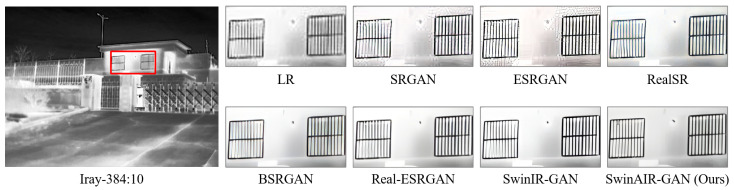
Visual comparison results achieved by different methods for real infrared images on the Iray-384 dataset [58] at the ×4 scale. LR represents the original image in the red box of the leftmost image.

**Figure 17:**
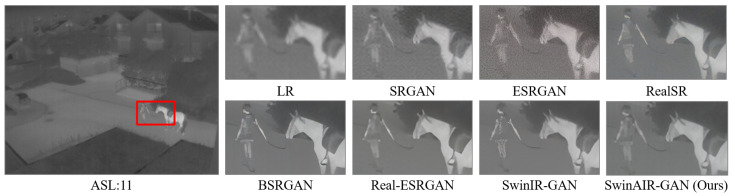
Visual comparison results achieved by different methods for real infrared images on the ASL-TID dataset [57] at the ×4 scale. LR represents the original image in the red box of the leftmost image.

Visual comparison. We compare the visual reconstruction results of different methods for real infrared images on multiple datasets at the ×4 scale. Compared with other methods, our proposed SwinAIR-GAN is able to restore details and textures more accurately and achieve better visual effects closer to the real images. For example, in Figure 15, only SwinAIR-GAN restores texture details consistent with the real scene. Other methods either introduce a lot of noise (e.g., ESRGAN [41], RealSR [54]) or generate incorrect details and textures (e.g., Real-ESRGAN [14], BSRGAN [13]). In other scenarios, our method recovers texture details that are more visually accurate and consistent with the LR image (e.g., the restoration of window lines on the left side in Figure 16 and the restoration of the person’s arm in Figure 17).

#### 4.4.2. Ablation Study

We conduct further ablation experiments on SwinAIR-GAN to validate the effectiveness of the dropout operation and the artifact discrimination loss. The models are retrained on the DV2K [54] dataset and reevaluated on the self-built dataset at the ×4 scale. We quantitatively assess the experimental results under custom degradation (using PSNR (dB)/SSIM as reference metrics) and unknown degradation (using NIQE/PI as reference metrics).

Effectiveness of the dropout operation. The dropout operation included in the SwinAIR-GAN lead to improved performance across all evaluation metrics compared with the same model without the dropout operation. The specific results can be found in Table 8. For real infrared images, the dropout operation results in an NIQE reduction of 0.2964 and a PI reduction of 0.1173. Furthermore, there are significant improvements in PSNR and SSIM under various degradations.

Effectiveness of the artifact discrimination loss. The SwinAIR-GAN incorporating the artifact discrimination loss exhibits lower NIQE/PI scores in the real infrared SR task and higher PSNR (dB) /SSIM scores in the custom-degraded SR task than the model without. The detailed results are shown in Table 9.

## 5. Conclusions

In this study, we devise a novel method, SwinAIR, for infrared image SR reconstruction. Through our carefully designed RSTAB, SwinAIR is capable of extracting both low- and high-frequency information from infrared images, enabling effective resolution enhancement. Building on this foundation, we further explore the SR reconstruction task for real infrared images by integrating SwinAIR with the U-Net to create SwinAIR-GAN. SwinAIR-GAN expands the degradation space to better simulate the process of real infrared image degradation. In addition, spectral normalization, dropout operation, and artifact discrimination loss are added to further enhance its performance. To better evaluate the reconstruction effects of different methods on real infrared images, we build an infrared data acquisition system. This system captures corresponding LR and HR infrared images of the same scene, addressing the lack of reference images in real infrared image SR tasks. Extensive comparative experiments on various datasets demonstrate the effectiveness of SwinAIR and SwinAIR-GAN. Although our method achieves superior performance and delivers visually striking results, there may still be differences between the reconstruction images by SwinAIR-GAN and the real infrared images due to the lack of high-quality infrared datasets [65]. Recent studies have shown that transfer learning can effectively improve network performance under the scarcity of datasets [45,66,67]. In the future, we will consider employing this technique, as well as expanding the training dataset, to further explore new methods for the real infrared image SR reconstruction task.

## Figures and Tables

**Table 1:** Detailed information about the convolutional layers in the discriminator network.

Name	Input Channels	Output Channels	Kernel Size	Stride
Conv-1	3	64	3	1
Conv-2	64	128	3	2
Conv-3	128	256	3	2
Conv-4	256	512	3	2
Conv-5	512	256	3	1
Conv-6	256	128	3	1
Conv-7	128	64	3	1
Conv-8	64	64	3	1
Conv-9	64	64	3	1
Conv-10	64	1	3	1

**Table 2:** Detailed parameters of the infrared data acquisition system.

Camera	Model	Resolution	Focal Length	Horizontal Field of View	Vertical Field of View
Iray	M3384012Y01312X	384 × 288	13mm	20°	15°
Jing Lin Chengdu	RTD6122C	640 × 512	18mm	14.8°	10.8°

**Table 3:** Quantitative comparison results achieved by different methods on the CVC-14 [56], Flir [57], Iray-384 [58], Iray-ship [59], Iray-aerial photography [60], and Iray-security [61] datasets. We use PSNR (dB)/SSIM * as the evaluation metrics. Params represents the parameters of the methods. Bold and underlined numbers indicate the best and second-best results, respectively.

Scale	Method	Params	CVC14	Flir	Iray-384	Iray-Security	Iray-Ship	Iray-Aerial Photography
×2	Bicubic	-	39.57/0.9570	32.23/0.7633	29.47/0.8980	33.09/0.9114	34.42/0.9287	32.37/0.9263
	SRCNN [5]	0.1M	39.93/0.9597	32.60/0.7834	31.06/0.9205	34.21/0.9291	35.17/0.9417	34.36/0.9480
	EDSR [7]	43.0M	42.53/0.9666	33.17/0.7930	32.16/0.9314	35.56/0.9383	36.51/0.9492	35.71/0.9586
	SRMD [63]	15.1M	43.62/0.9726	34.40/0.8193	33.14/0.9363	36.36/0.9439	37.67/0.9539	36.74/0.9618
	SRMDNF [63]	15.1M	43.77/0.9730	34.45/0.8202	33.32/0.9380	36.62/0.9454	37.82/0.9548	36.92/0.9630
	SRFBN [62]	3.5M	43.96/0.9736	34.53/0.8214	33.66/0.9409	37.25/0.9484	**38.10**/0.9560	37.21/0.9643
	SwinIR [10]	11.5M	**44.02**/**0.9737**	34.53/0.8208	33.77/0.9418	37.22/0.9484	37.98/0.9563	37.30/**0.9648**
	HAT [12]	20.8M	42.02/0.9701	33.10/0.7964	32.35/0.9320	36.64/0.9468	36.67/0.9504	36.60/0.9610
	SwinAIR (Ours)	9.2M	43.97/**0.9737**	**34.55**/**0.8221**	**33.81**/**0.9420**	**37.40**/**0.9488**	**38.10**/**0.9568**	**37.34**/**0.9648**
×3	Bicubic	-	36.68/0.9274	30.43/0.6947	27.03/0.8324	30.82/0.8575	31.68/0.8807	29.32/0.8596
	SRCNN [5]	0.1M	37.59/0.9352	30.92/0.7172	28.10/0.8601	31.72/0.8803	32.51/0.8982	30.76/0.8911
	EDSR [7]	43.0M	39.29/0.9444	31.45/0.7286	28.85/0.8774	32.57/0.8943	33.68/0.9090	31.78/0.9110
	SRMD [63]	15.3M	40.30/0.9530	32.63/0.7626	29.97/0.8840	33.61/0.9043	34.79/0.9157	32.95/0.9167
	SRMDNF [63]	15.3M	40.43/0.9535	32.69/0.7637	30.06/0.8859	33.70/0.9054	34.87/0.9167	33.04/0.9181
	SRFBN [62]	3.5M	40.81/0.9559	32.84/0.7662	30.38/0.8925	34.06/0.9099	**35.18**/0.9206	33.36/0.9228
	SwinIR [10]	11.5M	**40.87**/**0.9562**	32.84/0.7669	30.42/0.8932	33.97/0.9100	35.15/0.9203	33.37/0.9230
	HAT [12]	20.8M	39.94/0.9442	31.59/0.7285	29.50/0.8779	32.91/0.8974	33.94/0.9103	32.45/0.9146
	SwinAIR (Ours)	9.3M	40.85/0.9561	**32.85**/**0.7673**	**30.49**/**0.8939**	**34.15**/**0.9108**	**35.18**/**0.9212**	**33.38**/**0.9233**
×4	Bicubic	-	34.34/0.8985	29.19/0.6432	25.71/0.7817	29.83/0.8213	30.22/0.8463	27.70/0.8060
	SRCNN [5]	0.1M	35.09/0.9049	29.62/0.6645	26.50/0.8069	30.36/0.8409	30.84/0.8620	28.76/0.8358
	EDSR [7]	43.0M	36.84/0.9212	30.18/0.6813	27.14/0.8280	31.10/0.8592	31.20/0.8754	29.74/0.8642
	SRMD [63]	15.5M	37.87/0.9322	31.38/0.7199	28.29/0.8372	32.20/0.8725	33.20/0.8844	30.89/0.8725
	SRMDNF [63]	15.5M	37.98/0.9333	31.40/0.7210	28.36/0.8405	32.34/0.8750	33.21/0.8859	30.99/0.8755
	SRFBN [62]	3.5M	38.34/0.9367	31.57/0.7249	28.63/0.8483	32.55/0.8792	33.46/0.8905	31.24/0.8812
	SwinIR [10]	11.5M	38.38/0.9372	31.54/0.7251	28.58/0.8482	32.52/0.8794	33.46/0.8907	31.22/0.8815
	HAT [12]	20.8M	37.39/0.9173	30.02/0.6986	27.47/0.8283	31.49/0.8635	32.01/0.8811	30.61/0.8720
	SwinAIR (Ours)	9.3M	**38.52**/**0.9380**	**31.64**/**0.7272**	**28.78**/**0.8512**	**32.66**/**0.8811**	**33.51**/**0.8917**	**31.33**/**0.8835**

* Higher metrics mean better performance.

**Table 4:** Efficiency comparison results achieved by different methods. FLOPs (G) and inference speed (ms) * are used as metrics for evaluating computational complexity and real-time performance, respectively.

Scale	Image Size	Method	FLOPs (G)	Inference Speed (ms)
×4	64×64	EDSR [7]	205.83	10
		SRFBN [62]	530.93	22
		SwinIR [10]	49.28	32
		HAT [12]	82.04	77
		SwinAIR (Ours)	37.51	42

* Lower metrics mean better performance.

**Table 5:** Comparison results of using different types of pooling layers and different numbers of branches within the HFEL.

Number of Branches	Avgpool + Linear	Maxpool + Linear	PSNR *(dB)	SSIM *
1	✗	✓	38.19	0.9361
1	✓	✗	38.20	0.9364
2	✓	✗	38.52	0.9380
2	✗	✓	38.51	0.9379
2	✓	✓	37.96	0.9326

* Higher metrics mean better performance.

**Table 6:** Quantitative comparison results achieved by different GAN-based methods on the self-built, Iray-384 [58], and ASL-TID [57] datasets. BI and BD denote the degradation modes using bicubic downsampling and Gaussian blur downsampling, respectively. We use PSNR (dB)/SSIM * as the evaluation metrics. Params represents the parameters of the methods. Bold and underlined numbers indicate the best and second-best results, respectively.

Scale	Method	Loss Function	Self-Built Dataset (BI)	Self-Built Dataset (BD)	Iray-384 (BI)	Iray-384 (BD)	ASL-TID (BI)	ASL-TID (BD)
×4	BSRGAN [13]	$L_{1}+L_{\mathrm{perceptual}}+L_{\mathrm{adversarial}}$	31.98/0.8706	31.62/0.8663	25.58/0.7669	25.47/0.7773	33.26/0.8750	32.87/0.8703
	Real-ESRGAN [14]	$L_{1}+L_{\mathrm{perceptual}}+L_{\mathrm{adversarial}}$	30.94/0.8630	30.25/0.8513	25.10/0.7681	24.66/0.7703	32.66/0.8787	32.06/0.8704
	SwinIR-GAN [10]	$L_{1}+L_{\mathrm{perceptual}}+L_{\mathrm{adversarial}}$	29.91/0.8234	29.67/0.8210	24.64/0.7548	24.59/0.7674	32.87/0.8768	32.53/0.8725
	SwinAIR-GAN (Ours)	$L_{1}+0.1L_{\mathrm{artifact}}+L_{\mathrm{perceptual}}+L_{\mathrm{adversarial}}$	**33.47**/**0.9106**	**32.93**/**0.9043**	**25.89**/**0.7841**	**25.86**/**0.8037**	**33.82**/**0.9059**	**33.30**/**0.8992**

* Higher metrics mean better performance.

**Table 7:** Quantitative comparison results achieved by different GAN-based methods on the self-built, Iray-384 [58], and ASL-TID [57] datasets. We perform 4× SR reconstruction on the HR images in the dataset to validate the reconstruction performance of different methods on unknown degradation. NIQE/PI * are used as the evaluation metrics. Params represents the parameters of the methods. Bold and underlined numbers indicate the best and second-best results, respectively.

Scale	Method	Self-Built Dataset	Iray-384	ASL-TID
×4	BSRGAN [13]	6.3307/5.8951	4.3722/4.4544	5.7031/**5.1755**
	Real-ESRGAN [14]	6.2912/6.0504	4.4617/4.3883	6.1816/5.6404
	SwinIR-GAN [10]	6.4183/6.0457	4.0101/4.0049	**5.2140**/**4.9301**
	SwinAIR-GAN (Ours)	**6.0089**/**5.6015**	**3.9406**/**3.9722**	5.4822/4.9886

* Lower metrics mean better performance.

**Table 8:** Comparison results of whether use dropout operation or not.

Dropout	Unknown Degradation (NIQE/PI) ^1^	BI Degradation (PSNR (dB)/SSIM) ^2^	BD Degradation (PSNR (dB)/SSIM) ^2^
✗	6.3053/5.7188	32.46/0.8904	32.22/0.8924
✓	6.0089/5.6015	33.47/0.9106	32.93/0.9043

^1^ Lower NIQE/PI mean better performance. ^2^ Higher PSNR (dB) /SSIM mean better performance.

**Table 9:** Comparison results of whether use artifact discrimination loss or not.

Dropout	Unknown Degradation (NIQE/PI) ^1^	BI Degradation (PSNR (dB)/SSIM) ^2^	BD Degradation (PSNR (dB)/SSIM) ^2^
✗	6.6458/6.0230	32.67/0.8969	32.37/0.8934
✓	6.0089/5.6015	33.47/0.9106	32.93/0.9043

^1^ Lower NIQE/PI mean better performance. ^2^ Higher PSNR (dB) /SSIM mean better performance.

## Data Availability

The original contributions presented in the study are included in the article, further inquiries can be directed to the corresponding author.
